# Male tobacco smoke load and non-lung cancer mortality associations in Massachusetts

**DOI:** 10.1186/1471-2407-8-341

**Published:** 2008-11-24

**Authors:** Bruce N Leistikow, Zubair Kabir, Gregory N Connolly, Luke Clancy, Hillel R Alpert

**Affiliations:** 1 Department of Public Health Sciences, University of California, Davis, 1 Shields Avenue, Davis, CA 95616-8638, USA; 2 Harvard School of Public Health, Division of Public Health Practice, 401 Park Drive, Landmark Center, 3rd Floor (East), Boston MA 02215, USA; 3 Research Institute for A Tobacco Free Society, the Digital Depot, Thomas Street, Dublin 8, Ireland

## Abstract

**Background:**

Different methods exist to estimate smoking attributable cancer mortality rates (Peto and Ezzati methods, as examples). However, the smoking attributable estimates using these methods cannot be generalized to all population sub-groups. A simpler method has recently been developed that can be adapted and applied to different population sub-groups. This study assessed cumulative tobacco smoke damage (smoke load)/non-lung cancer mortality associations across time from 1979 to 2003 among all Massachusetts males and ages 30–74 years, using this novel methodology.

**Methods:**

Annual lung cancer death rates were used as smoke load bio-indices, and age-adjusted lung/all other (non-lung) cancer death rates were analyzed with linear regression approach. Non-lung cancer death rates include all cancer deaths excluding lung. Smoking-attributable-fractions (SAFs) for the latest period (year 2003) were estimated as: 1-(estimated unexposed cancer death rate/observed rate).

**Results:**

Male lung and non-lung cancer death rates have declined steadily since 1992. Lung and non-lung cancer death rates were tightly and steeply associated across years. The slopes of the associations analyzed were 1.69 (95% confidence interval (CI) 1.35–2.04, r = 0.90), and 1.36 (CI 1.14–1.58, r = 0.94) without detected autocorrelation (Durbin-Watson statistic = 1.8). The lung/non-lung cancer death rate associations suggest that all-sites cancer death rate SAFs in year 2003 were 73% (Sensitivity Range [SR] 61–82%) for all ages and 74% (SR 61–82%) for ages 30–74 years.

**Conclusion:**

The strong lung/non-lung cancer death rate associations suggest that tobacco smoke load may be responsible for most prematurely fatal cancers at both lung and non-lung sites. The present method estimates are greater than the earlier estimates. Therefore, tobacco control may reduce cancer death rates more than previously noted.

## Background

Estimation of mortality caused by tobacco use is an essential basis for state, national, and international tobacco control efforts. Cancer mortality, which accounts for an estimated annual 1.4 million or one third of, deaths globally, due to smoking [1], is of particular importance. Recent estimates of smoking-attributable cancer mortality utilize lung cancer mortality data as an indicator of the accumulated hazards of tobacco smoking and tightly link several temporal, ethnic, and geographic cancer mortality disparities to smoking [1,2]. However, those indirect estimates have at least two flaws. In the authors' words, the Peto estimates are based on a "simple halving of the excess risk [linked to smoke exposure which] is obviously not a satisfactory procedure, for it is crude and arbitrary and may seriously underestimate some of the true hazards of tobacco" [3]. And both estimates are based in part on the Cancer Prevention Study (CPS) – II, which has considerable selection, exposure misclassification, and other biases [3-5]; and they may seriously underestimate the actual smoking attributable mortality. Further, the absolute death rates among the non-smokers and the smokers (and thus the smoker/nonsmoker death rate ratios) in the CPS-II study cannot be generalized over the US population [3,6].

A simpler method for computing cumulative tobacco smoke damage has recently been introduced. This method utilizes the associations between lung cancer ("smoke-load") and non-lung cancer death rates across all cancer sites. The smoke-load method may be more representative, less biased, and provide better population-specific smoking attributable fractions (SAFs) than prior methods [7-9].

Since Peto's method was introduced in the 1990s [3], several cancers have been found to be tobacco-related. These include some of the most common cancers around the world such as cancers of the stomach, liver, uterine cervix, kidney (renal cell carcinoma), and myeloid leukemia. In addition, the cancer risks of tobacco smoking when combined with exposure to other known carcinogens are greatly enhanced for some cancer sites ().

In summary, different methods exist to estimate smoking attributable cancer mortality rates (Peto and Ezzati methods, as examples). However, the smoking attributable estimates using these methods cannot be generalized to all population sub-groups. A simpler method has recently been developed that can be adapted and applied to different population sub-groups. The smoke-load method has been applied to different populations at other time periods [6-9]. In this study, we assessed cumulative tobacco smoke damage (smoke load)/non-lung cancer mortality associations across time from 1979 to 2003 among all Massachusetts males and ages 30–74 years.

## Methods

The present analysis excludes certain groups with variations in cancer death rates due to causes other than smoking in order to increase the sensitivity to detect associations between smoking rates and non-lung cancer death rates. Non-lung cancer deaths refer to death rates from cancers other than lung cancer. Factors other than smoking that are known to cause variations in cancer mortality rates are: a) changes in female pharmacological estrogen and anti-estrogen treatments, mammography, or hysterectomy; b) child cancer treatment changes; c) stomach cancer mortality declines from 1930 to approximately 1979 associated with historical changes in environmental factors such as Helicobacter Pylori infection; d) historical changes in the classification of general causes of death such as changes in the International Classification of Diseases (ICD) and changes associated with improved cancer biopsy rates and classification associated with the 1970s Medicare and 1971 National Cancer Acts; and g) less diagnostic specificity at older ages.

Each of the above factors are less likely to substantially affect more recent triennial cancer death rates among middle-aged males, a population with high smoking prevalence and low cause of death misclassification rates [10]. Therefore, annual and triennial time-series rates among males aged 30–74 years during the calendar years since initiation of ICD 9^th ^revision use in 1979 are analyzed in this study. Non-lung-cancer death rates were determined by subtracting lung-cancer rates from all sites cancer rates.

Published mortality rates per 100,000 from the National Center for Health Statistics [11,12] were used adjusted to the 2000 US Standard Million population. Lung cancer death rates were used as a smoke load bioindex [7]. Linear regression analyses were performed with lung and non-lung cancer rate as the independent and dependent variable, respectively.

Smoking attributable fractions (SAF) and their sensitivity range (SR) were calculated using the formula SAF = 1 – (estimated unexposed cancer death rate)/(observed rate) based on the observed lung/non-lung cancer death rate relationship and 95% confidence intervals (CI) and estimated all sites cancer death rate among unexposed persons of 63 (SR 43 to 93) per 100,000. The unexposed rates used are, respectively, the recent cancer death rates observed among male South Asian Californians [13], female Asian Indian Americans excluding breast, cervix, endometrial, and ovary (female-specific) cancers, [13]. The estimates based on the linear association of Massachusetts male lung/non-lung cancer death rates across the years 1979–1988 and 1995–2001 (when prostate cancer death misclassification was rare) and the lung cancer death rate observed among United States White male "never-smokers" (16.8 lung cancer deaths/100,000 referenced in Cancer Prevention Study II [CPS]) were multiplied by 0.75, which is the minimum reduction required to adjust the rate from the CPS II age standard to the 2000 US age standard population [14].

Cancer death rates in the unexposed male population, aged 30–74 years, were estimated by multiplying the all-age male cancer death rates by 0.95, which is equal to the ratio of ages 30–74 years/all ages Massachusetts male all sites cancer death rates averaged across 2002–2003 per the US Centers for Disease Control, Wide-ranging OnLine Data for Epidemiologic Research system (CDC WONDER) [15].

## Results

**Figure 1:**
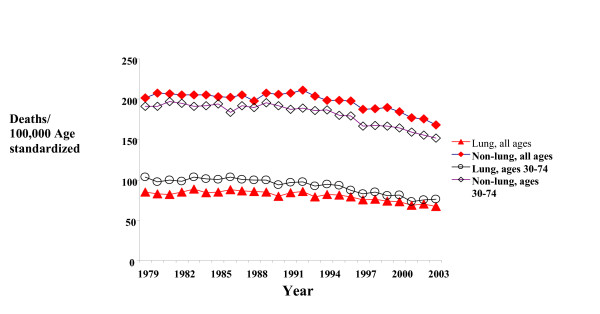
Male lung and non-lung cancer death rates by year and years of age in Massachusetts.

**Figure 2:**
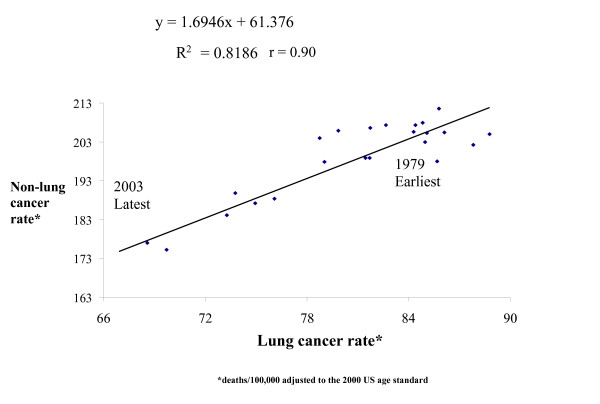
Scatter plot of male lung versus non-lung cancer death rates (earliest points and latest points) in Massachusetts (line segments connect consecutive years).

**Figure 3:**
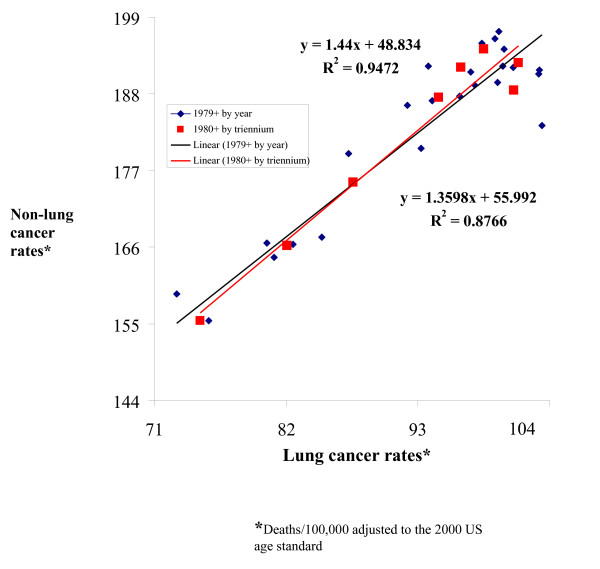
Scatter plot of male lung cancer death rates versus all other cancer death rates at ages 30–74 years (earliest and latest points) and from 1980 to 2003 by triennium in Massachusetts (line segments connect consecutive years).

Massachusetts male cancer death rates peaked in the early 1990s, including the rates for all sites, and non-lung cancer death rates for all age groups and for the 30–74 years age group (Figure 1). A plot of the annual lung cancer death rates against their corresponding non-lung cancer death rate reveals a strong lung/non-lung cancer death rate association among males of all ages and an even stronger association among males aged 30–74 years (Figures 2 and 3). The slopes of these associations are 1.69 (95% CI 1.35–2.04, r = 0.90), or 1.36 (CI 1.02–1.96, r = 0.95) when adjusted for possible autocorrelation) and 1.36 (CI 1.14–1.58, r = 0.94) without detected autocorrelation (Durbin-Watson statistic = 1.8), respectively.

Figure 3 also shows a scatterplot of the triennial rates from 1980–2003, the slope of which is 1.44 (CI 1.10–1.78, r = 0.97) without detected autocorrelation (Durbin-Watson statistic = 1.98). The lung/non-lung cancer death rate associations suggest that year 2003 all-sites male cancer death rate SAFs are 73% (SR 61–82%) across all ages and 74% (SR 61–82%) for males aged 30–74 years, or 196 deaths per 100,000.

## Discussion

The strong lung/non-lung cancer death rate associations observed among Massachusetts males suggest that tobacco smoke load is a potential cause of most prematurely fatal cancers in this population. These associations suggest that all-sites cancer death rate SAFs are 73% (SR 61–82%) for males over all ages and 74% (SR 61–82%) for males aged 30–74 years.

SAFs of age-adjusted cancer death rates calculated by this methodology are substantially higher than the cancer death rate SAFs calculated based on the CPS II relative risks. The present method incorporates all non-lung cancers; sizeable, representative, recent male populations; and age-adjusted death rates, which are the most reliable measure of progress in global action against cancer [16]. In contrast, previous calculations of SAF were generally based on sometimes outdated lists of smoking-related cancer sites [1,17]; tobacco smoke exposure based on smoking status, which extensively underestimates or misclassifies due to brief, unrecognized, disregarded, unrecalled or secondhand smoke exposure [4,18]; as well as select smaller, racially homogeneous populations, that are unrepresentative of the general population or even of Whites only [5,6,19].

The limitations of this study are noted. Extrapolation from population-level associations has previously resulted in inaccurate estimates of individual relative risk due to the ecologic fallacy [20]. The results of these analyses may be unrepresentative of the association among females, in other US states, or of longer-term trends prior to ICD-9. The associations observed do not distinguish between types of smoking exposure due to in-utero, other secondhand smoke, sensitive stage (teenage) [21], or active smoking since smoke load reflects cumulative lifetime damage [22].

The validity of the present findings is reinforced by the strong and consistently positive smoke load/cancer death associations seen over time among both males of all ages and aged 30–74 years. In an earlier analysis among Black US males, lung cancer death rates predicted approximately 98% and 97% of the variances in non-lung cancer death rates throughout the 34% rise from 1969–1990 and the subsequent decline of 11% from 1990–2000, respectively [8]. The present analysis of more recent, and more representative data, including comprehensive smoke load estimation, concurrent with outcome assessments is in contrast to the use of less recent and less-representative data reported in most cohort studies to date [4,5,19] and less reliable and comprehensive point exposure measures. Those measures are often based on single smoking self-report observations that were assessed as long as decades before the measured outcomes [22].

SAFs can be inaccurate and misleading under various circumstances such as omission of certain smoking-attributable cancers, use of unrepresentative relative risks, misclassified exposure status, and incomplete disclosure of assumptions concerning sensitivity. SAFs that are based on site-specific cancer death relative risks assume that smoking causes no cancer deaths at other cancer sites. The error in this assumption has been shown repeatedly in subsequent studies linking smoking to additional cancer sites [23]. Artificially low relative risk estimates might result from high smoke exposures and death rates among "never smokers" in the cohort or from unrepresentatively low exposures and death rates among "smokers" due to low prior smoking or accelerated quitting among smokers informed of the risk of premature death.

Each of the above issues appears to be present in the CPS II cohort and the smoke effect estimates that are based on it. The CPS II definition of "lifelong never smoker" likely included many irregular, brief, or forgetful smokers and only excluded persistent, regular smokers (persons had who smoked at least one cigarette per day for one year) [24]. Most CPS II "smokers" resurveyed at twelve years denied current smoking [4]. This might help to explain the reason that concurrent national average male death rates greatly exceeded the average CPS II male death rates for all causes [4] and exceeded even the CPS II "smoker" lung cancer death rate at ages 35–49 years [7,15,24].

The smoke load/non-lung cancer death rate ratios observed in this study are remarkably consistent across time. Smoke load variation provides a likely and possibly causal explanation for a large majority of the cancer death rate disparities studied to date (see results) [7,9,13]. Lung cancer death rates (smoke load) can explain 88% of the variance in non-lung-stomach-uterine corpus rates from 1985 to 2004 among Korean females [9]. The estimated Korea female all-sites cancer death rate SAF in 2004 was 43% (sensitivity range 29–56%) [9]. Residual confounding from smoke load variations [22] may be an explanation for associations found in prior studies between cancer deaths and other epidemiological risk factors, such as cooking or outdoor air pollution, diets, industrial exposures, or medical treatments, made independently of smoking status or other poor proxies for smoke load [25-29]. Alternatively, those non-tobacco exposures may theoretically cause premature lung and non-lung cancer deaths in the same ratio as does smoke exposure.

Nonetheless, the temporal trends of lung cancer epidemics in most countries seem to be more compatible with smoking patterns than with cooking, other air pollution, oral tobacco, or nutrition-driven epidemics. Other than declining death rates attributable to effective treatments for uterine, breast and cervical cancer, most currently available cancer treatments may have as little effect on smoke loads and premature mortality as treatments for premature mortality due to HIV, Kaposi's sarcoma, and pneumocystosis had before anti retrovirals were found that reduced HIV loads [30].

## Conclusion

In conclusion, the finding of consistent, strong associations observed between smoke load and most cancer mortality and the large SAFs [7] stresses the significance of increasing support for tobacco control research, science, and policy. Further research is needed to develop biochemical measures of individual smoke load, study cancer disparities among larger racially diverse populations, and examine the reasons for the varying degrees of association observed between lung cancer and other cancer death rates among various populations [7,9,13]. Until such validated robust biochemical measures for individual tobacco load are developed or refined, simpler and less biased methods such as the smoke load method can be adapted and applied to different population sub-groups. Nonetheless, the improved ability to assess the population effect of tobacco smoke damage can be used to better inform cancer control policy including the regulation of tobacco products and smoking in public places.

## Competing interests

The authors declare that they have no competing interests.

## Authors' contributions

BNL conceived the study design, ZK, LC and GNC secured funding, ZK liaised with the UC Davis and supervised the study; BNL contributed to data collection and performed the analyses, with contributions from ZK and HA; BNL, ZK and HA prepared the original draft manuscript, with inputs from LC and GNC. The funding agency had no role in the study design, interpretation of results or decision to publish. All the authors contributed to the manuscript writing and have approved the final version. BNL is the guarantor of this paper.

## Pre-publication history

The pre-publication history for this paper can be accessed here:


